# Bioconcentration and Metabolism of Emodin in Zebrafish Eleutheroembryos

**DOI:** 10.3389/fphar.2017.00453

**Published:** 2017-07-11

**Authors:** Jiefeng Chen, Shaodong Li, Mengping Liu, Christopher Wai Kei Lam, Zheng Li, Xinjun Xu, Zuanguang Chen, Wei Zhang, Meicun Yao

**Affiliations:** ^1^School of Pharmaceutical Sciences, Sun Yat-sen University Guangzhou, China; ^2^Third Affiliated Hospital of Sun Yat-sen University Guangzhou, China; ^3^State Key Laboratory of Quality Research in Chinese Medicine, Macau Institute for Applied Research in Medicine and Health, Macau University of Science and Technology Macau, China

**Keywords:** emodin, anthraquinones, bioconcentration, metabolism, zebrafish eleutheroembryos

## Abstract

Emodin is a major active anthraquinone of various herbal laxatives, which can exert many pharmacological effects. However, chronic use of anthranoid laxatives, even at low dosages, may cause melanosis coli (MC). It has been suggested that the accumulation of anthraquinones is a risk factor in the MC process. To investigate the accumulation of emodin, we conducted a bioconcentration study of emodin in zebrafish eleutheroembryos. Based on the economic cooperation and development (OECD) 305 test, zebrafish eleutheroembryos were exposed to emodin at a constant concentration for 48 h, before the test media were replaced by the blank medium for 24 h of depuration. To eliminate the effect of metabolism of emodin for assessment of the bioconcentration factor (BCF), we also conducted a modified test for which zebrafish eleutheroembryos were exposed to the non-renewed test media, whose emodin concentration decreased with time. At different exposure time points, zebrafish eleutheroembryos and exposure media were sampled for analysis of emodin concentration using HPLC-MS/MS. The results showed rapid accumulation of emodin in zebrafish eleutheroembryos to reach a steady-state concentration within 24 h. Meanwhile, emodin was actively metabolized by zebrafish eleutheroembryos to result in 29.5–40.7% of its elimination. In the groups with high or low concentrations of emodin, the standardized BCF (sBCF) values in the standard test were 24.0 and 20.0, while those in the modified test were 50.4 and 52.0. These results showed that emodin could accumulate in zebrafish eleutheroembryos when used for 48 h and beyond, suggesting that the accumulation of anthraquinones may be a risk factor in the MC process. Accordingly, emodin should be unsuitable for long-term use due to its accumulation.

## Introduction

Emodin, an active anthraquinone in herbal laxatives such as aloe, senna, cascara sagrada, semen cassia, and rhubarb, can exert various pharmacological effects, including laxative, anti-allergic, anti-inflammatory, anti-cancer, and anti-diabetic activities (Liu et al., 2012). Moreover, it can promote weight loss through its laxative property (Matsuda et al., 2008; Liu et al., 2010), and products containing laxative herbs (like cassia seed tea) are widely used for long-term weight loss. However, its safety also should be concerned. Previous studies have shown its hepatotoxicity and renal toxicity due to its cytotoxic effect via apoptosis by affecting the mitochondria (Li et al., 2012; Cui et al., 2014). Besides, the disturbance effect to glutathione and fatty acid metabolism in liver cells also is the reason of its hepatotoxicity (Liu et al., 2015). Some other studies have reported its reproductive toxicity, showing that it can disrupt the expression of testicular genes and inhibit human sperm function (Oshida et al., 2011; Luo et al., 2015). Its genotoxicity and DNA-damaging properties also have been found by *in-vitro* study (Li et al., 2010). Furthermore, many case reports (Han et al., 2010; Kav and Bayraktar, 2010) have shown that chronic use of anthranoid laxatives and weight-loss products may cause melanosis coli (MC), which can increase the risk of colonic neoplasm (van Gorkom et al., 1999). This suggests that the safety of long-term use of emodin should be carefully considered, in agreement with the conclusion from previous studies (Dong et al., 2016).

MC is a brownish pigmentation of the colon caused by the accumulation of dark brown pigments in macrophages of the lamina propria. It is considered that anthraquinones can induce the apoptosis of colonic epithelial cells, which furtherly induces MC (Byers et al., 1997; Chen et al., 2011). Previous study showed that this apoptosis was associated with the up-regulated expression of Bcl-2 (Zhang et al., 2013). Emodin as an anthraquinone derivative also has the same activity of induction of apoptosis (Ma and Li, 2014), therefore its potential toxicity of MC should be concerned. However, the exact mechanism is still unclear, because other factors can also contribute to MC pathogenesis.

Bioconcentration is the process that chemical substances accumulate in organisms from the ambient environment only through breath and direct contact (Arnot and Gobas, 2006). The degree of the bioconcentration can be expressed as the bioconcentration factor (BCF), which is defined as the ratio between the concentration of the chemical in the organism and environment at steady state. Polycyclic aromatic hydrocarbons (PAHs), like naphthalene and anthracene, are a kind of hard-degraded substances with high BCF (Kuhnert et al., 2013). Since anthraquinones are derivatives of anthracene, they may have some properties similar to PAHs. The potential risk from the accumulation of anthraquinones should not be neglected. Considering the fact that long-term use of low-dosage anthraquinone laxative (like weight-loss products) may induce MC, we assumed that the accumulation of anthraquinones in colonic cells is a risk factor in the MC process.

Zebrafish is a small tropical fish that has been used widely as a model organism for toxicology studies. Seventy percent of the genes of zebrafish are similar to those of humans (Howe et al., 2013), making zebrafish extremely appropriate for elucidating the pharmacology of medicines. Its eleutheroembryos are considered to be non-animal systems (Halder et al., 2010) meeting the 3R principles of ethical research (replacement, refinement, and reduction), which has been applied as an alternative model for estimation of bioconcentration factor (Augustine-Rauch et al., 2010; Chng et al., 2012). The economic cooperation and development (OECD) bioconcentration test 305 is a common protocol to estimate BCF in fish (OECD, 1996, 2012). Based on the OECD 305, the BCF is expressed as the ratio between the uptake and depuration constants of the chemical (El-Amrani et al., 2013). However, the BCF value of the chemical may be undervalued when calculated by the general method without taking into account of the metabolism of the chemical (Kuhnert et al., 2013). When the metabolism of the test chemical in the organism is significant, the BCF value should be corrected for avoiding underestimation accumulation of the chemicals. However, this is difficult to achieve with the classical model in the OECD 305.

To investigate the potential accumulation of emodin, the bioconcentration of emodin was studied using zebrafish eleutheroembryos. Considering the fact of that emodin is prone to be metabolized, a modified bioconcentration model was performed to calculate the metabolism rate of emodin thereby correcting the underestimation of the BCF value of emodin. For analyzing the concentrations of emodin in zebrafish eleutheroembryos and exposure medium, a validated HPLC-MS/MS methodology was developed.

## Materials and methods

### Reagents and test samples

Reference standards of emodin and puerarin (internal standard, IS) were purchased from National Institute for the Control of Pharmaceutical and Biological Products (Beijing, China). For quantitative analysis, stock solutions of emodin and IS were prepared in methanol at a concentration of 88.4 and 66.5 μg/mL, respectively. For the bioconcentration study, a stock solution of emodin was prepared in DMSO at a concentration of 2 mg/mL. All stock solutions were stored at 4°C and diluted daily to working solutions for experiments. HPLC grade methanol was purchased from SK Chemicals (Ulsan, Korea). Deionized water with a resistivity of 18.2 MΩ was produced by a Milli-Q water purification unit (Barnstead International Inc., Dubuque, Iowa, USA).

### HPLC-MS/MS conditions

HPLC was performed on a Shimadzu LC-20AD series HPLC system (Kyoto, Japan) consisting of two LC-20AD pumps, a CTO-20A column oven (set at 35°C) and a SIL-20A autosampler. Analyte separation was achieved on a Phenomenex Luna C_18_ column (150 × 2.00 mm, 5 μm) preceded by a Phenomenex C_18_ security guard column, with an isocratic mobile phase consisting of 0.1% formic acid and methanol (20:80, v/v) at a flow rate of 0.3 mL/min.

MS detection was performed on a Thermo Finnigan TSQ quantum mass spectrometer (San Jose, CA, USA). Ultra-high pure nitrogen was used as the sheath and auxiliary gas. The ESI source was operated in negative ion mode under the following conditions: spray voltage, 3.5 kV; capillary temperature, 400°C; sheath gas, 35 arbitrary units; auxiliary gas, 15 arbitrary units. For quantification in multiple-reaction monitoring (MRM) cone voltage, collision energy and precursor to production transition m/z for emodin and IS were as shown in Table 1. For metabolism study of emodin, three phase II metabolites of emodin were monitored simultaneously using MRM mode (Teng et al., 2007).

**Table 1:** MS/MS parameters for detection of analytes and IS.

	**Parent ion (m/z)**	**daughter ion (m/z)**	**Cone voltage (v)**	**Collision energy (eV)**
**COMPOUND**				
Emodin	269	225	30	29
Puerarin (IS)	415	225	25	28
**METABOLITES**				
Sulfated emodin (M1)	349	269	30	40
Glucosylated emodin (M2)	431	269	30	40
Glucuronidated emodin (M3)	445	269	30	40

### Bioconcentration experiments

#### Preparation for zebrafish eleutheroembryos and exposure media

Zebrafish embryos at 6 h post fertilization (hpf) were supplied by the Zebrafish Model Animal Facility, Institute of Clinical and Translational Research, Sun Yat-sen University, China. The embryos were maintained in egg water (10% Hank's solution, 13.7 mM NaCl, 0.54 mM KCl, 0.025 mM Na_2_HPO_4_, 0.044 mM KH_2_PO_4_, 0.13 mM CaCl_2_, 0.1 mM MgSO_4_, and 0.42 mM NaHCO_3_) for development. At 72 hpf, hatched eleutheroembryos with no malformation were select for bioconcentration experiments. Exposure media were prepared with egg water and final conditions of resulting exposure solution were: temperature 26 ± 2°C, dissolved oxygen ≥60% saturation and pH 7.0 ± 0.2, based on the OECD guideline. In addition, the process of embryo development and exposure tests were both conducted under a 12:12 h (light:dark) photoperiod. In this study, the loading rate of eleutheroembryos ranged between 0.3 and 0.5 g/L following OECD recommendation. The studies were approved by the Animal Ethics Committee of Sun Yat-sen University and followed the Guide for Care and Use of Laboratory Animals published by the US National Institute of Health (US-NIH, 2011).

The nominal concentrations of the test substance were selected according to the requirements given by the OECD test 305, with the concentration below 1% of its LC_50_ value. In a previous study (He et al., 2012), after exposure to 1.5 μg/mL of emodin for 48 h, the mortality rate of zebrafish embryos was lower than 50%. Therefore, the high concentration selected for the bioconcentration experiments was 15 ng/mL and a lower level was set at 7.5 ng/mL, half of the high level.

#### Exposure test at constant concentration

Based on the OECD 305 and previous studies (El-Amrani et al., 2012, 2013; Tu et al., 2014), the standard test was applied to obtain the BCF of emodin. Zebrafish eleutheroembryos were divided into three groups and transferred individually in three tanks: one for control (containing no emodin) and two for test groups containing the media with different concentrations of emodin. The test process included uptake period (0–48 h) and depuration period (48–72 h). During the uptake period, zebrafish eleutheroembryos were constantly exposed to the test media, which was renewed every 12 h to guarantee a constant exposure concentration. At 48 h of exposure, the exposure media were replaced by the egg medium, followed by a 24-h depuration period. The exposure media and eleutheroembryos were sampled at 0, 2, 6, 12, 24, 36, 48, 50, 52, and 72 h. At each time point, three replicate samples of 20 eleutheroembryos and 0.2 mL sample of media were collected, respectively. Sampled eleutheroembryos were rinsed three times with deionized water. All samples were stored at −80°C until analysis.

#### Exposure test at variable concentration

To investigate the metabolic rate constant, zebrafish eleutheroembryos were exposed to emodin at a certain initial concentration for a modified test. Compared with the standard test, the major characters of the modified test were that that the test medium was no longer refreshed and its concentration decreased with time. Bioaccumulation experiment was performed in four tanks, two for controls (at different concentrations without the addition of eleutheroembryos) and another two contained eleutheroembryos and emodin at different concentrations. Specifically, the ratio of the number of eleutheroembryos and the volume of medium was defined as “fish-to-water ratio” which was kept at 1:1 (fish/mL) in the whole experiment. The exposure media and eleutheroembryos were sampled at 0, 2, 6, 12, 24, 30, 36, 48, and 72 h of the exposure time. At each time point, three replicate samples of 20 eleutheroembryos and 0.2 mL of exposure media were collected (from controls and exposure tanks). Subsequently, a total of 60 mL of exposure media was removed from each tank to make the fish-to-water ratio constant. Collected eleutheroembryos were rinsed three times with the cold deionized water. All samples were stored at −80°C until analysis.

### Analytical procedures

#### Preparation of the exposure media

Medium of 200 μL was mixed with 50 μL IS methanol solution (100 ng/mL) and extracted with 1 mL ethyl acetate by vortex mixing for 3 min. After centrifugation at 16,000 rpm for 10 min, the organic phase was collected and dried with nitrogen. The residue was reconstituted with 200 μL mobile phase and centrifuged at 16,000 rpm for 10 min. Twenty microliters of supernatant was injected into the HPLC-MS/MS system for analysis.

#### Extraction of emodin in zebrafish eleutheroembryos

This method was adapted from a previously developed method for analyzing of emodin in rat plasma (Huang et al., 2012) and pesticide in zebrafish eleutheroembryos (El-Amrani et al., 2012). A sample of 20 eleutheroembryos was freeze-dried and weighted, then spiked with 100 μL IS methanol solution, and suspended in an ultrasonic bath (40 kHz, 400 W) for 15 min. The eleutheroembryos suspension was further extracted ultrasonically with 300 μL of methanol for 15 min. The mixture was centrifuged at 16,000 rpm for 10 min, and 20 μL of the supernatant was injected into the HPLC-MS/MS system for analysis of emodin.

### Toxicokinetic model and data analysis

#### Constant exposure concentration model for the standard test

A first-order, one-compartment bioconcentration model was used to describe the uptake and depuration processes of emodin:

(1) $$\begin{array}{r} \frac{dC_{f}}{dt}=k_{1}C_{w}-k_{2}C_{f}\;\left(\text{uptake}\right)\;\frac{dC_{f}}{dt}=-k_{2}C_{f}\;\left(\text{deputration}\right) \end{array}$$

where *C*_*f*_ (ng/g) and *C*_*w*_ (ng/mL) are the concentrations of the target compounds in zebrafish eleutheroembryos and exposure solution at a given time *t* (h), respectively, and *k*_1_ (L/h/kg dry weight) and *k*_2_ (1/h) are the toxicokinetic uptake and depuration rate constants, respectively. Assuming that at *t* = 0 the concentration of test chemical in the organism is zero and the concentration of chemical in the exposure medium is constant, Equation (2) is obtained:

(2) $$\begin{array}{r} C_{f}=\frac{k_{1}}{k_{2}}C_{w,0}\left(1-{\text{e}}^{-k_{2}t}\right)\;\left(\text{uptake}\right)C_{f}=C_{f,0}e^{-k_{2}t}\;\left(\text{deputration}\right) \end{array}$$

where *C*_*w*_,_0_ is the exposure concentration and *C*_*f*_,_0_ is the chemical in the organism at the beginning of the depuration process. The BCF can be calculated from the ratio of *k*_1_ and *k*_2_. When the steady state is reached, with the *C*_*f*_ and *C*_*w*_ constant, Equation (3) can be obtained.

(3) $$\begin{array}{r} C_{f}/C_{w}=BCF_{k}=k_{1}/k_{2} \end{array}$$

Equations (2), (3) are the general formulas for bioconcentration study from the OECD 305. Specifically, the apparent deputation is the sum of elimination, fish growth, metabolic transformation and fecal egestion. As the wet weights of zebrafish eleutheroembryos change slightly and the digestive tract of them has not developed fully, the fish growth and the fecal egestion can be ignored. Therefore, the constitute of *k*_2_ can be described as follow (Kuhnert et al., 2013):

(4) $$\begin{array}{r} k_{2}=k_{m}+k_{out} \end{array}$$

in which *k*_*m*_ (1/h) is the metabolism rate constant and *k*_*out*_ (1/h) is the elimination rate constant in zebrafish eleutheroembryos. For unstable compounds which are easily metabolized, *k*_*m*_ is not ignorable but hard to obtain from Equation (2).

#### Modified model for the modified test

When the exposure concentration is not kept constant and decreases mainly due to the factors beyond the bioconcentration, a more complex model need to be used. Assuming first order kinetics, the bioconcentration process can be described as follow (Andreu-Sanchez et al., 2012):

(5) $$\begin{array}{rcl} \frac{dC_{f}}{dt} & = & k_{1}C_{w}-k_{2}C_{f}\;\left(\text{fish}\right) \end{array}$$

(6) $$\begin{array}{rcl} \frac{dC_{w}}{dt} & = & -\lambda C_{w}\;\left(\text{medium}\right) \end{array}$$

where *k*_1_, *k*_2_, *C*_*w*_, and *C*_*f*_ are the same as the corresponding parameters in Equation (1) and λ (1/h) is the dissipation rate constant of the concentration of the compound in the exposure medium. Assuming that at *t* = 0 the concentration of test chemical in the organism is zero, Equations (7), (8) are obtained:

(7) $$C_{f}=\{\begin{array}{ccc} \frac{C_{w,0}k_{1}\left(e^{-\lambda t}-e^{-k_{2}t}\right)}{k_{2}-\lambda },\;\;if\;k_{2} & \neq & \lambda \\ C_{w,0}k_{1}te^{-\lambda t},\;if\;k_{2} & = & \lambda \end{array}$$

(8) $$\begin{array}{r} C_{w}=C_{w,0}e^{-\lambda t} \end{array}$$

where the *C*_*w*_,_0_ is the initial concentration of test chemical in the medium. When the steady state is reached, Equation (9) is obtained:

(9) $$C_{f}/C_{w}=BCF_{k}=\underset{t\to \infty }{\lim}\left(\frac{C_{f}}{C_{w}}\right)=\{\begin{array}{c} \frac{k_{1}}{k_{2}-\lambda },if\;k_{2}>\lambda \\ +\infty ,\;if\;k_{2}\leq \lambda \end{array}$$

Equation (7) is the modified form of Equation (2). Only if the exposure concentration is constant, Equation (7) is equivalent to the uptake phase of Equation (2). The dissipation of test chemical in the medium is concerned as sum of physical, chemical and biological processes:

(10) $$\begin{array}{r} \lambda =k_{p}+k_{c}+k_{b}=k_{p}+k_{c}+\left(k_{b-m}+k_{b-c}+k_{b-d}\right) \end{array}$$

where *k*_*p*_ is from solvent evaporation and analyte absorption on the container wail, *k*_*c*_ is mainly from oxidation, hydrolysis and photolysis, and *k*_*b*_ is mainly from metabolism (*k*_*b*−*m*_), bioconcentration (*k*_*b*−*c*_) and biodegradation (*k*_*b*−*d*_). *k*_*p*_ and *k*_*c*_ are constant under certain conditions and can be obtained from control group. *k*_*b*−*m*_ is affected by density of fish. The higher the density of fish (fish-to-water ratio, number of fish per unit volume medium) is, the bigger the *k*_*b*−*m*_ is. *k*_*b*−*c*_ is variable and proportional to the negative derivative of natural logarithmic concentration-time curve of test chemical accumulation in the organism. *k*_*b*−*d*_ is from the microorganism in the medium, which can be consider as constant. When the bioconcentration is the only factor highly affects the exposure concentration, the λ is not constant and the dissipation process can be described as follow:

(11) $$\begin{array}{r} \frac{dC_{w}}{dt}=R_{f-w}\left(k_{2}C_{f}-k_{1}C_{w}\right) \end{array}$$

where *R*_*f*−*w*_ (g/L) represents the mass of fish per unit volume of water.

In this study, as the weights of zebrafish eleutheroembryos were very low, the bioconcentration could hardly affect the *C*_*w*_. With the fish-to-water ratio kept unchanged, λ was unchanged and equal to the sum of *k*_*p*_, *k*_*c*_, *k*_*b*−*m*_, and *k*_*b*−*d*_. Therefore, Equations (5), (6) were appropriate for the modified test, ignoring the bioconcentration.

#### Data analysis

Values of *k*_1_, *k*_2_, and λ were estimated by non-linear regression using the software OriginPro v.8.5 (OriginLab, Northampton, MA, USA). For comparison to the previous study, the standardized BCF for wet weight was calculated based on the dry weight percentage of about 20% found for the eleutheroembryos (Petersen and Kristensen, 1998).

## Results and discussion

### Analytical performance of emodin analysis

Our method provided recoveries of 87.0–90.5% range for eleutheroembryos suspension and 97.7–101.4% for medium, respectively. No matrix effect was observed, which were assessed by comparing the responses achieved from post-extraction blank sample and mobile phase spiked with the analyte. The inaccuracies ranged from −3.4–2.9% to −2.3–4.6% for eleutheroembryos suspension and medium, respectively. The precisions represented as RSD were not more than 8.6 and 8.8% for eleutheroembryos suspension and medium, respectively. Calibration curves were plotted from 5 to 7 concentration points (0.5–150 ng/mL for eleutheroembryos suspension and 0.25–50 ng/mL for medium). The instrumental limits of detection (LODs), calculated as a signal to noise ratio of three, were 0.2 ng/mL for eleutheroembryos suspension and 0.1 ng/mL for the exposure medium.

### Bioconcentration, metabolism, and toxicokinetics of emodin in zebrafish eleutheroembryos

Zebrafish eleutheroembryo is a powerful tool applied to various aspects of toxicity studies such as acute toxicity, teratogenicity, toxicokinetecs, toxicodynamics, adverse effects, and long-term effects (Scholz et al., 2008). A number of studies have provided evidences that eleutheroembryo as an alternative to adult fish can greatly be applied to bioconcentration study (Wiegand et al., 1999; Schreiber et al., 2009; El-Amrani et al., 2012; Tu et al., 2014). Compared with adult fish, one of the major characters of eleutheroembryos is that eleutheroembryos develop depending on its own yolk consumption instead of external feeding (Halder et al., 2010). Generally, it is considered that the eleutheroembryos cannot feed freely until 139.5 hpf at 26°C breeding temperature (Straehle et al., 2012). According to the definition, the process of the bioconcentration comprises breath and direct contact but the uptake in the diet is not included (Arnot and Gobas, 2006); i.e., only the passive diffusion should be taken into account. Considering the character of eleutheroembryos, the use of eleutheroembryos can avoid the feeding-induced extra intake of test chemicals in long-term exposure test. Moreover, the poor digestive system of eleutheroembryos ensure that the excretion slightly affects the depuration of test chemicals. Therefore, the bioconcentration in eleutheroembryos can be completely considered as a passive process, which greatly conforms the definition of bioconcentration. Our study also confirmed the applicability of zebrafish eleutheroembryos on bioconcentration study.

**Figure 1:**
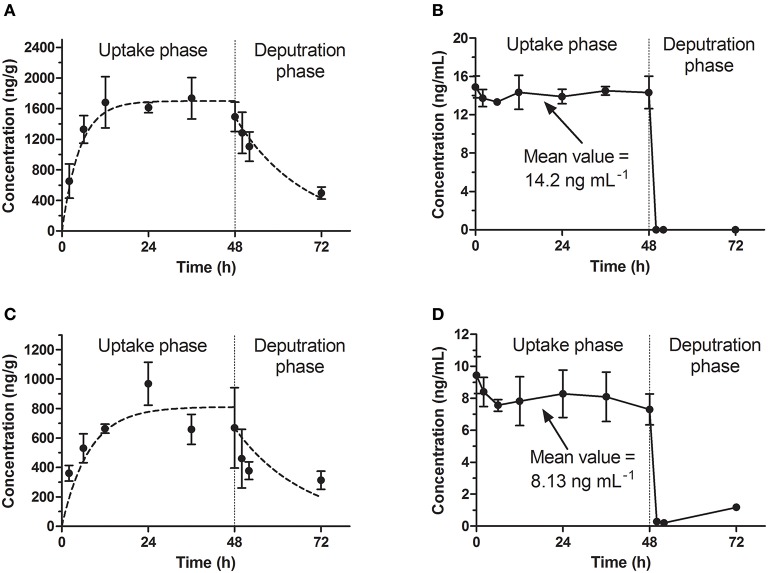
Bioconcentration of emodin in the standard test. **(A,C)** The concentration-time profiles in eleutheroembryos at high and low level concentrations. **(B,D)** Emodin concentration of exposure medium at high and low level concentrations.

**Figure 2:**
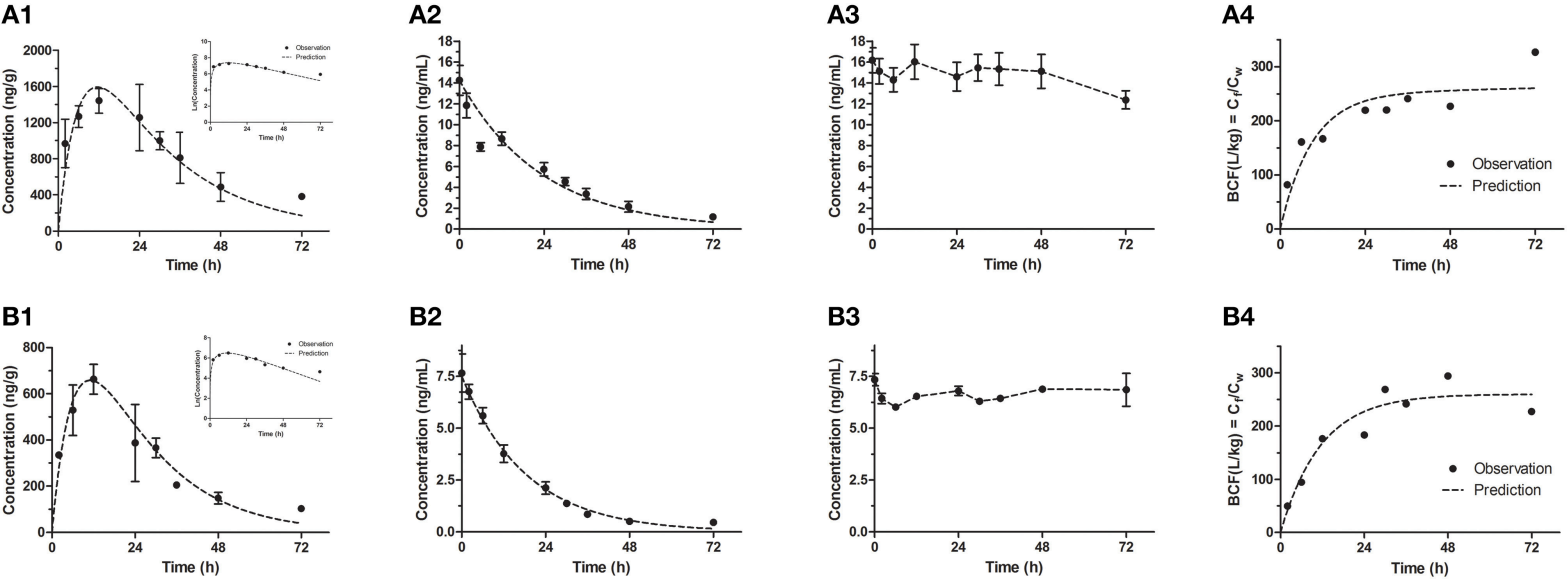
Bioconcentration of emodin in the modified test for high **(A1–A4)** and low **(B1–B4)** concentrations. **(A1,B1)** The concentration-time profiles in eleutheroembryos, with the natural logrithmic concentration-time profiles in the upper right corner; **(A2,B2)** Concentration-time profiles of exposure medium; **(A3,B3)** Concentration-time profiles of control groups; **(A4,B4)** Residues (C_f_/C_w_) in eleutheroembryos exposed to the emodin.

In our study, the results of bioconcentration process of emodin in zebrafish eleutheroembryos are shown in Figure 1 for the standard test, and Figure 2 for the modified test. In both tests, emodin obviously accumulated in the bodies of the eleutheroembryos.

In the standard test, the toxicokinetics of different concentration groups were similar. The concentrations of emodin in exposure media (hereinafter referred to as C_w_) were considered to be constant (the variations of concentrations were below 10%) with the values (mean ± *SD*) of 14.2 ± 0.5 and 8.13 ± 0.70 ng/mL for high and low concentration groups respectively. A slight decrease of the analyte concentration was observed, which was likely due to the metabolism and possibly some degree of adherence on the walls of the tanks. The accumulation profiles of emodin in zebrafish eleutheroembryos are shown in Figures 1A,C. The concentrations of emodin in zebrafish eleutheroembryos (hereinafter referred to as C_f_) increased gradually with the exposure time and then reached plateau at 24 h, which suggested that the bioconcentration process reached steady state after only 24 h of exposure. The concentrations at plateau in the high concentration group were nearly double of that in the low concentration group. In the depuration phase, the zebrafish lavae were transferred to blank medium to eliminate the residual emodin, whose concentrations were decreased by about 50% within 24 h.

In the modified test, the toxicokinetic process was remarkably different from the standard test. As the exposure media were not renewed, C_w_ were not constant and decreased by over 90% within 72 h, with the initial concentrations of 14.2 ± 1.42 and 7.66 ± 0.92 ng/mL for two concentration groups, respectively. No obvious variations of concentration were observed in control groups, which showed that emodin was stable under the test environment. In other words, the physical factors, chemical factors and the biodegradation slightly affected the C_w_ and could be ignored. On the other hand, since the mass of emodin in the eleutheroembryos' bodies was below 3% of total mass of emodin in the tanks, we thought that the influence of the bioconcentration on C_w_ could be ignored. Meanwhile, we concluded that the decrease of C_w_ was mainly due to the metabolism. In the bodies of eleutheroembryos, C_f_ increased gradually at first with peak concentrations obtained at 12 h of exposure, and then decreased continuously with the decline of C_w_. The real-time BCFs, calculated by the ratios of C_f_ and C_w_, increased with time and reached plateau after 24 h of exposure, suggesting that the distribution had reached steady state.

The toxicokinetic parameters are shown in Table 2. The symbols *k*_1_ and *k*_2_ designate the uptake and depuration rate constants, respectively, obtained from Equations (2), (7). *k*_1_ and *k*_2(bioc)_ were determined in exposure process, and *k*_2(dep)_ was from depuration phase in the standard test. The dissipation rate constants (λ) of emodin in media were obtained from Equation (8). BCF values were obtained from Equations (3), (9). BCF_ss_ values were the experimental values at steady state, which were calculated as the ratio between C_f_ and C_w_ at the maximum time of exposure time (48 h for the standard test and 72 h for the modified test). BCF_k_ values were obtained from parameters *k*_1_, *k*_2_ and λ, which showed that BCF values in the standard test were lower than those in the modified test, due to the apparent metabolism of emodin.

**Table 2:** Toxicokinetic parameters and BCF values of emodin obtained from zebrafish eleutheroembryos.

**con.^a^ (ng/mL)**	**k_1_^b^ (L/kg/h)**	**k_2(upt)_ (1/h)**	**k_2(dep)_ (1/h)**	**λ (1/h)**	**BCF_k_^c^**	**BCF_ss_^d^**	**sBCF^e^**	**cBCF^f^**	**clog D_ow_^g^ (pH = 7)**
**Test 1**								33.0	2.30
15	24.4	0.204	0.0519	–	120	104	24.0		
7.5	13.2	0.132	0.0506	–	100	91.6	20.0		
**Test 2**									
15	28.1	0.154	–	0.0427	252	326	50.4		
7.5	22.4	0.140	–	0.0540	259	227	52.0		

a *The con. were the nominal concentrations instead of the measured concentrations*.

b *k_1_ was the uptake rate constant. k_2(upt)_ and k_2(dep)_ were the depuration rate constants obtained in uptake phase and depuration phase, respectively. λ was dissipation rate of the medium concentrations*.

c *BCF_k_ was the fitted BCF with the toxicokinetic equations*.

d *BCF_ss_ was the measured BCF at steady state, whose values were the ratios between C_f_ and C_w_ at the maximum time of exposure time (48 h for the standard test and 72 h for the modified test)*.

e *sBCF was the standardized BCF (or wet-weight BCF), whose values were generally one fifth of BCF_k_*.

f *cBCF was the calculated BCF based on the QSAR model*.

g *clog D_ow_ was the calculated log Dow using Advanced Chemistry Development (ACD/Labs) Software V11.02, obtained from SciFinder®(https://scifinder.cas.org/)*.

In previous studies, several quantitative structure-activity relationship (QSAR) models have been established to calculate the BCF (cBCF) of organic compounds. These QSAR models showed that the BCF values of the chemicals can be correlated with n-octanal/water partition coefficients (*K*_ow_) of the chemicals. A classical equation has been established for the BCF_L_ of lipophilic substances in zebrafish eleutheroembryos (Petersen and Kristensen, 1998):

(12) $$\begin{array}{r} \text{log }BCF_{L}=0.64+0.92\text{ log }K_{ow} \end{array}$$

Herein, for ionizable chemicals, apparent oil-water partition coefficients (*D*_*ow*_) rather than *K*_*ow*_ are used (Ding et al., 2016). The *D*_*ow*_ of chemicals change with pH, which also called pH-corrected *K*_*ow*_. In this study, the pH of medium was 7 and did not change with the concentration of emodin. To compare with the estimated values, the BCF values obtained in this study were standardized to wet-weight BCF (sBCF), by transferring the dry-weight concentration to wet-weight concentration. sBCF represents the real accumulation level of chemicals in alive organism rather than in dry organism. Generally, for zebrafish eleutheroembryos, sBCF is one fifth of dry-weight BCF.

**Figure 3:**
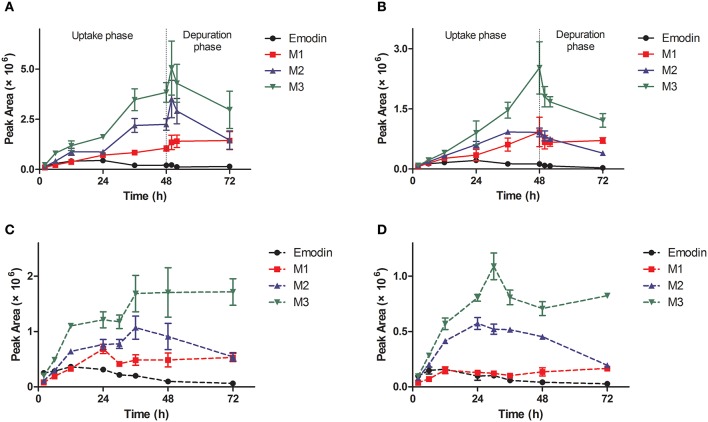
Profiles of metabolites of emodin in zebrafish eleutheroembryos in the standard test **(A,B)** and the modified test **(C,D)** at high and low concentrations. M1, sulfated emodin; M2, glucosylated emodin; M3, glucuronidated emodin.

The metabolites of emodin were monitored by MRM mode. The previous study has proved that phase II metabolism is the major way of elimination of emodin *in vivo*. In our work, three classical phase II metabolites, mono-sulfate (M1), mono-glucosylate (M2), and mono-glucuronide (M3) were chosen for this study. Both M1 and M2 are common metabolites of emodin in human (Liu et al., 2012), while M3 is a specific metabolite existing in zebrafish eleutheroembryos (Hu et al., 2012). In zebrafish eleutheroembryos, the metabolites of emodin accumulated with increased exposure time in all tests (Figure 3). In the uptake phase of the standard test, the concentrations of metabolites increased by a linear trend, which indicated that the formation rates of the metabolites could be considered as approximately constant. In the depuration phase of the standard test, the metabolites concentration decreased with time but more than half of the metabolites remained in the body after 24-h depuration. Under the microscope, it was observed that emodin and its metabolites were accumulated mainly in the intestines of eleutheroembryos (Supplementary Material 1). At 72 h of test time, the color of the intestinal contents was still deep, while it became light after an extra 24-h depuration. In the modified test, the accumulation rates of metabolites were fast at first and turned slow subsequently. These results showed that emodin could be highly metabolized *in vivo*, which is in agreement with the previous studies performed in mammals (Teng et al., 2007; Liu et al., 2012). No metabolite was detected in the media probably due to their low concentrations that were below the low limit of detection. More interestingly, di-conjugates were also detected in zebrafish eleutheroembryos, which maybe contributed by the fact that two metabolic reactions (any two of the glucuronidation, glucosylation, and sulfation processes) can be induced by the active phenolic hydroxyl groups in emodin (Hu et al., 2012). These results further implicated that emodin can be highly metabolized. Since the mono-conjugate is the main form of metabolites of emodin in the human body (Liu et al., 2012), no further study on di-conjugates was performed in our work.

### Comparison of two bioconcentration models and derivation for the calculation of metabolism rate constant

**Figure 4:**
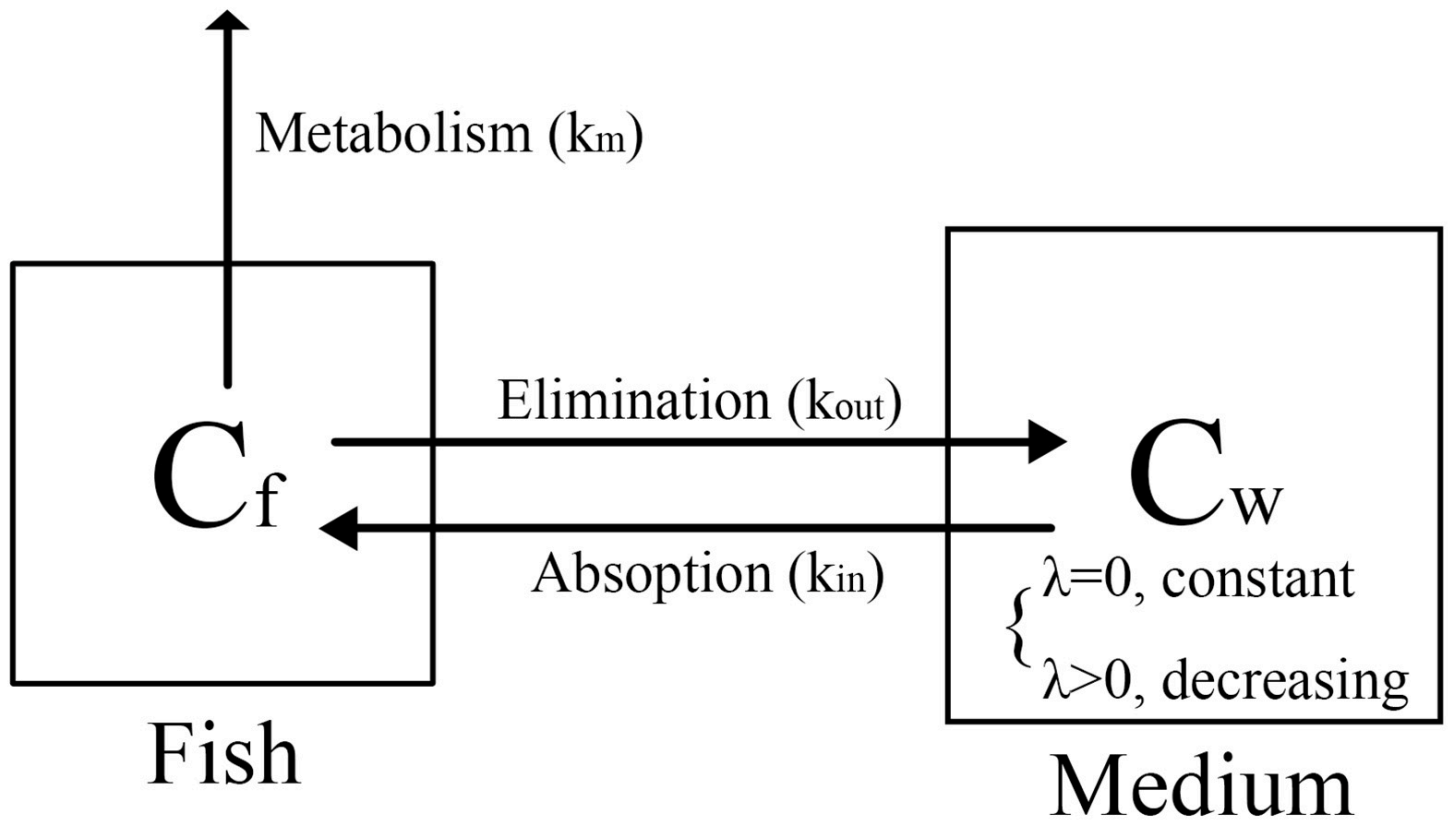
Proposed distribution process in zebrafish eleutheroembryos.

The bioconcentration processes of the two tests are illustrated in Figure 4. These processes comprised uptake (*k*_*in*_), elimination (*k*_*out*_) and metabolism (*k*_*m*_), where *k*_*in*_ is equal to *k*_1_, and the sum of *k*_*out*_ and *k*_*m*_ is equal to *k*_2_.

The standard test is a bioconcentration experiment preformed at constant exposure concentration, which can be regarded as a long-term exposure model or a repeated exposure model. Therefore, the process of the standard test is simple and we just need to focus on the emodin concentration in zebrafish eleutheroembryos. In this model, *k*_2_ is considered as the *k*_*out*_ with the *k*_*m*_ assumed as zero. For the calculation of BCF of emodin, since the high metabolism was not included, *k*_*out*_ was overestimated, leading the BCF value being lower than the actual value.

The modified test is a bioconcentration experiment preformed at a certain initial exposure concentration, which can be considered as a single exposure model. The relations among *k*_1_, *k*_2_, *k*_*in*_, *k*_*out*_, and *k*_*m*_ are the same as those in the standard test. In this study, during 0–12 h of the exposure time, the concentration process was the same as the standard test, thus the concentration-time profiles for zebrafish eleutheroembryos were similar to those of the standard test. Only at 12 h, *C*_*f*_ reached a peak value, and the uptake rate was equal to the sum of elimination rate and metabolism rate (Equation 13).

(13) $$\begin{array}{r} V_{in}=V_{out}\;+\;V_{m}\;\to \;k_{int}C_{w}=k_{out}C_{f}\;+\;k_{m}C_{f},\text{ only at }12\text{ h} \end{array}$$

Since *k*_*m*_ was not zero, the uptake rate (*V*_*in*_) was higher than the elimination rate (*V*_*out*_). In the other word, for the exposure medium, the amount of the lost emodin was more than that of the reuptake of emodin, leading the *C*_*w*_ decreasing continually. The decrease of *C*_*w*_ caused the uptake rate (*k*_*int*_*C*_*w*_) to decline, further causing the decrease of C_f_ at 12–72 h of exposure time. Within 24–72 h of exposure time, a steady state was reached with the BCF tending to be constant (Figures 2A4,B4). During this period, the natural logarithmic concentration-time curve of C_f_ of the modified test was approximately linear (Figures 2A1,B1), which indicated that the internal concentration decreased with an unchanged rate constant. Here, we defined this period as “depuration period” of the modified test, with a depuration rate constant *k*_3_ (1/h). The *k*_3_ could be obtained by linear fitting the Equation (14), and its values was the slope of this equation.

(14) $$\begin{array}{r} LnC_{f}=-k_{3}t+b \end{array}$$

During the depuration period, the process of *C*_*f*_ followed Equation (15), derived from Equations (4), (5):

(15) $$\begin{array}{r} \frac{dC_{f}}{dt}=-k_{3}C_{f}=k_{int}C_{w}-k_{out}C_{f}-k_{m}C_{f} \end{array}$$

From Equations (4), (9), the following equation could be obtained:

(16) $$\begin{array}{r} BCF=\frac{k_{int}}{k_{out}+k_{m}-\lambda }=\frac{C_{f}}{C_{w}} \end{array}$$

From Equations (15), (16), Equation (17) could be obtained:

(17) $$\begin{array}{r} k_{3}=\lambda \end{array}$$

Equation (17) further supports our claim that the decrease of C_f_ was directly attributed to the decrease of the exposure concentration. However, the observed values of *k*_3_ at 0.0251 and 0.0284 in the high and low concentration groups respectively were obviously lower than the values of λ, which were probably due to the intestinal accumulation of emodin. When the decrease of C_w_ contributed to a decline in C_f_, the intestinal emodin was released into the whole body, providing a buffer for slowing down the decrease rate of C_f_. This hypothesis can be confirmed by the fact that the values of *k*_2_ in depuration phase was lower than that in uptake phase in the standard test (Table 2). In this case, the bioconcentration process of emodin may be fitted better with the two-compartments model.

The decrease of C_w_ was from uptake and elimination, expressed as Equation (18). It is important to note that as the uptake rate and elimination rate are the variables for C_f_, a conversion factor *R*_*f*−*w*_ (g/L), the mass of fish per unit volume of water, should be introduced into the equation. In our work, the average of the dry weight of one fish was 0.0609 ± 0.0076 mg. With the fish-to-water ratio kept at 1:1 (fish: mL), the *R*_*f*−*w*_ was calculated to be 0.0609 g/L.

(18) $$\begin{array}{r} -\lambda C_{w}=R_{f-w}\left(k_{out}C_{f}-k_{in}C_{w}\right) \end{array}$$

From Equations (15–18), Equation (19) could be derived. It is shown that the value of *k*_*m*_ can be obtained from λ, *R*_*f*−*w*_ and BCF. In other words, to calculate the *k*_*m*_, it was crucial to keep the *R*_*f*−*w*_ (or the fish-to-water ratio) constant in the whole test. Since *k*_*m*_, like *k*_*in*_ and *k*_*out*_, is a characteristic constant only related to the chemical reactivity of the substance and the animal species, the ratio of *k*_*m*_–*k*_2_ should be a fixed value. From Equation (19), values of *k*_*m*_ were calculated with the values of 0.0455 and 0.0570, which was 29.5 and 40.7% of the values of *k*_2_, for high and low concentration group respectively. Finally, it is concluded that the metabolism constituted 29.5–40.7% of the depuration process of emodin in zebrafish eleutheroembryos.

(19) $$\begin{array}{r} k_{m}=\lambda \cdot \left(1+\frac{1}{R_{f-w}\cdot BCF}\right) \end{array}$$

In the standard test the underestimated BCF values can be corrected by subtracting the *k*_*m*_ from *k*_2_ and the corrected BCF values were 169.6 and 168.6 (with the sBCF of 33.9 and 33.7) for high and low exposure level, respectively. In this study, *k*_*m*_ was approximately equal to λ because of very small value of $\frac{1}{R_{f-w}\cdot BCF}$. Based on Equation (9), the BCF in the modified test could be approximately considered as the corrected BCF. Nevertheless, with the intestinal accumulation, the values of BCF in the modified test were high than the corrected BCF.

About all, the modified test can be used for the calculation for the *km*. However, it is a special process only fitted to the chemicals which are easily biotransformed. For those depurated mainly with the unchanged form, only the standard method is suitable to study their BCF. Moreover, the form of the Equation (19) is variable and may be changed if the test chemicals are unstable in the medium. In these cases, more factors should be concerned (including physical, chemical and biological factors), but the method of derivation is similar as above.

### Potential risks of emodin and anthraquinones

In the previous study, it was showed that emodin has very poor oral bioavailability owing to its extensive glucuronidation in intestine and liver. In the gut, emodin is absorbed in the intestinal cells by passive diffusion and metabolized to phase II metabolites (Liu et al., 2012). It seems that the accumulation and the biotransformation of emodin in intestinal cells is similar to that in zebrafish eleutheroembryos. Although the half-life of emodin is not long (5.4 h in rats, Huang et al., 2012), emodin still can be locally accumulated in the intestinal cells by repeated intake, as the intestinal wall directly contacts to the ingested food. Since emodin is highly metabolized, its single dose cannot cause accumulation, as the process of the modified test. However, when the emodin used for a long time (for weight loss or chronic constipation), the intracellular concentration of emodin could be accumulate to a high level continually (dozens of times higher than the concentration in lumen), as the process of the standard test. Although, the emodin could be accumulated in the whole gut with it passing the intestine, the accumulation most likely occurred in the colon instead of other parts of the digestive tract, as the in-take food stays in colon for most of the time. Accordingly, as the alimentary canal toxicity of anthraquinones only occurred in the colon, it could be inferred the potential accumulation of anthraquinones may be a risk factor concerned with MC process. On the other hand, as the lipid content of zebrafish eleutheroembryos and intestinal cells are similar (Petersen and Kristensen, 1998; Trapp and Horobin, 2005), the accumulation level of substances in these two organisms are similar. In other words, the extent of accumulation of chemicals in intestinal cells can be estimated by the data from zebrafish eleutheroembryos. Since the estimated value with QSAR model was similar with the observed values in our study, it can be considered that this QSAR model can be applied to other anthraquinones. With the value of pH of colon lumen was about 6–7 (Nugent et al., 2001), the estimated BCF of typical anthraquinones are listed in Table 3, which shows that some kinds of anthraquinones have high BCF values. These illustrated that the emodin as well as other anthraquinones could highly accumulate in the colonic cells.

**Table 3:** Estimated values of BCF of typical anthraquinones.

	**pH** = **6**		**pH** = **7**	
	**clogD_ow_^a^**	**cBCF^b^**	**clogD_ow_**	**cBCF**
Emodin	3.34	258	2.30	33.0
Aloe-emodin	3.01	134	2.12	23.1
Rhein	1.37	5.23	0.49	0.915
Chrysophanol	4.55	2,840	3.80	643
Physcion	4.78	4,480	3.80	643
Obtusifoline	2.02	18.9	1.36	5.16

a *Clog Dow: calculated log Dow using Advanced Chemistry Development (ACD/Labs) Software V11.02, obtained from SciFinder®(https://scifinder.cas.org/)*.

b *cBCF was the calculated BCF based on the QSAR model*.

Moreover, the effect of intestinal flora should not be ignored. It was reported that the metabolites of emodin and combined emodin can be microbiologically converted to free emodin, and only the free emodin can be absorbed by intestinal cells (Moreau et al., 1985). When taking herbal laxative, the combined anthraquinones cannot be absorbed, owing to their high molecular weight and polarity, and finally reach to the colon (van Gorkom et al., 1999). In the colon, their aglycones can be released by beta-glucoside enzyme from resident microflora, and subsequently the free anthraquinones can be absorbed and function on the colon, which works synergistically with the bioconcentration effect, leading to a further increase of the intracellular concentration of anthraquinones.

It is considered that the apoptosis of colonic epithelial cells is the main mechanism of the anthraquinone-induced MC (Chen et al., 2011). As the targets of apoptosis activity of emodin are inside the cells (Dong et al., 2016), its toxicity is affected by the intracellular concentration rather than the exposure concentration. In the standard test, the test media were renewed every 12 h, which was similar as the repeated administration, while the modified test was similar as the single administration. Comparing the results of these two tests, it could be inferred that the long-term use of anthraquinones could make their concentrations in the intestinal cells remain at high level, while short-term use could not make it. The accumulation highly increases the risk of long-term use of anthraquinones, which may be a possible reason why only long-term use (instead of short-term use or a single high dose) of anthraquinones can cause MC. In conclusion, the accumulation of emodin as well as anthraquinones is a non-negligible risk factor of the MC process.

## Conclusions

In this study, emodin was found to accumulate in zebrafish eleutheroembryos with high BCF value, which supported our hypothesis that the accumulation of anthraquinones is potential risk factor of the MC process. It can be concluded that emodin can be easily metabolized in the body, but it is unsuitable for long-term administration. To correctly estimate the BCF of emodin, two tests were performed in parallel based on two models. With the *R*_*f*−*w*_ introduced into the modified model, we derived the formula for metabolism rate constant of emodin. Our work displayed an alternative method for correcting the BCF of the chemicals which are easy to be metabolized.

## Author contributions

MY and WZ conceived the experiments and helped to coordinate support and funding. JC performed the research and drafted the manuscript. SL, ML, XX, and ZC participated in the experiments. ZL and CL analyzed the data and edited the paper. All authors read and approved the final manuscript.

### Conflict of interest statement

The authors declare that the research was conducted in the absence of any commercial or financial relationships that could be construed as a potential conflict of interest.
