# Supplementary material for: Bioconcentration and Metabolism of Emodin in Zebrafish Eleutheroembryos
Source: Front Pharmacol. 2017 Jul 11;8:453. doi: 10.3389/fphar.2017.00453 (PMC5504456; doi:10.3389/fphar.2017.00453)

Supplementary Material

Bioconcentration and metabolism of emodin in zebrafish eleutheroembryos

Jiefeng Chen, Shaodong Li, Mengping Liu, Christopher Wai Kei Lam, Zheng Li, Xinjun Xu, Zuanguang Chen, Wei Zhang*, Meicun Yao*

*** Correspondence:**
These authors contributed equally to this work:
Wei Zhang
wzhang@must.edu.mo
MeiCun Yao
yaomeicun@gmail.com

1 Supplementary 1

The micrograph of zebrafish larvae in the standard test at 0 h **(A),** 24 h **(B),** 72 h **(C)**, 96 h **(D)** and 120 h **(E)**. Arrows: accumulation of emodin and its metabolites in intestine.

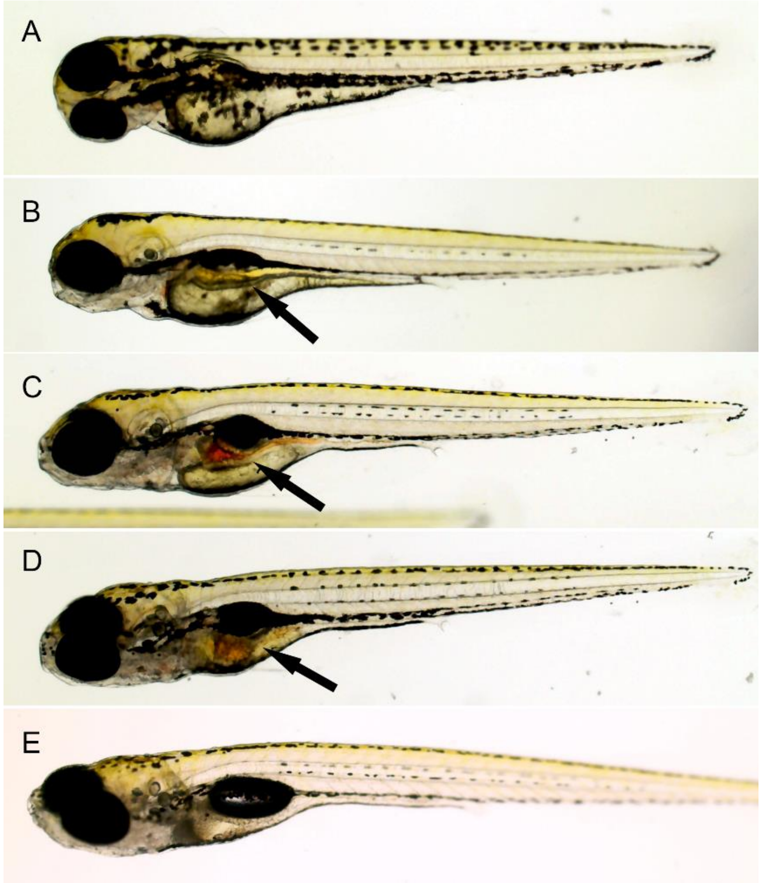

Supplement: Supplementary file 1 [file Image1.PDF]
